# Increased Serum Soluble Urokinase-Type Plasminogen Activator Receptor (suPAR) Levels in FSGS: A Meta-Analysis

**DOI:** 10.1155/2019/5679518

**Published:** 2019-04-04

**Authors:** Jiwon M. Lee, Jae Won Yang, Andreas Kronbichler, Michael Eisenhut, Gaeun Kim, Keum Hwa Lee, Jae Il Shin

**Affiliations:** ^1^Department of Pediatrics, Chungnam National University College of Medicine, Daejeon, Republic of Korea; ^2^Department of Nephrology, Yonsei University Wonju College of Medicine, Wonju, Gangwon, Republic of Korea; ^3^Department of Internal Medicine IV (Nephrology and Hypertension), Medical University Innsbruck, Innsbruck, Austria; ^4^Pediatric Department, Luton & Dunstable University Hospital NHS Foundation Trust, Luton, UK; ^5^Keimyung University College of Nursing, Daegu, Republic of Korea; ^6^Department of Pediatric Nephrology, Severance Children's Hospital, Seoul, Republic of Korea; ^7^Department of Pediatrics, Yonsei University College of Medicine, Seoul, Republic of Korea; ^8^Institute of Kidney Disease Research, Yonsei University College of Medicine, Seoul, Republic of Korea

## Abstract

**Introduction:**

The soluble urokinase-type plasminogen activator receptor (suPAR) has been found to be elevated in primary focal segmental glomerulosclerosis (pFSGS). However, its usefulness as a biomarker for FSGS remains controversial. We conducted a meta-analysis aiming at investigating the significance of suPAR in diagnosing pFSGS.

**Methods:**

Electronic databases (PubMed and EMBASE) were searched to identify studies comparing suPAR levels in FSGS patients and controls, from the earliest available date to May 1, 2018. A random-effects model with standardized mean difference (SMD) was used for meta-analyses. Risk of bias was assessed using the Newcastle-Ottawa quality assessment scale.

**Results:**

A total of 187 articles were screened, and the final analysis included 13 articles. In comparison to healthy controls, serum suPAR levels were significantly increased in pFSGS patients (SMD, 1.07, 95% confidence interval (CI) 0.65 to 1.48; participants = 814; studies = 9, *I* ^2^ = 85%). Higher suPAR levels were also found in patients with pFSGS compared to those with minimal change disease (SMD 0.53, 95% CI 0.22 to 0.84). Of note, such a difference was not found in pediatric groups (SMD 0.42, 95% CI -0.13 to 0.96) while it was more evidently noted in adult patients (SMD 1.32, 95% CI 0.90 to 1.74). Serum suPAR levels did not differ between pFSGS patients in remission compared to those in active proteinuric state (SMD 0.29, 95% CI -0.30 to 0.88). Comparison with membranous nephropathy and IgA nephropathy showed no significant difference.

**Conclusions:**

Our meta-analysis demonstrated that, in comparison to both healthy controls and controls with minimal change disease, suPAR levels were significantly higher in adult patients with pFSGS. suPAR levels did not differ between pFSGS patients during the initial period of diagnosis and those in remission.

## 1. Introduction

Primary focal segmental glomerulosclerosis (pFSGS) is a leading cause of glomerulonephritis which can progress to end-stage renal disease (ESRD). pFSGS is estimated to be responsible for 40% of adult and 20% of pediaric cases with nephrotic syndrome [1]. Prompt differential diagnosis of FSGS in a proteinuric patient is therefore an important step in the management and disease course. Efforts to discover potential novel biomarkers have been attempted to promote early diagnosis of pFSGS.

In the pathogenesis of pFSGS, circulating factors have been regarded as significant because in about 40% of patients, this disease recurs after transplantation [2, 3]. The soluble urokinase plasminogen activator receptor (suPAR) is a protein circulating in the human blood and body fluids, which is present at low concentration in healthy individuals and high levels in patients with infections, chronic kidney disease (CKD), and other inflammatory disorders [4]. In that, its usefulness as a biomarker has been investigated in various diseases, such as sepsis [5], pneumonia [6], chronic obstructive pulmonary disease (COPD) [7], and kidney diseases including pFSGS [3, 8–10]. Regarding pFSGS, suPAR has been proposed to be a marker for diagnosis and posttransplantation recurrence [1, 11–13]. However, its significance and reliability as a diagnostic marker for pFSGS has later been refuted and still remains controversial [14–16].

Hence, we conducted a meta-analysis of published studies that have measured suPAR in patients with pFSGS and controls, in order to investigate and review the usefulness of suPAR as a potential biomarker.

## 2. Methods

### 2.1. Literature Search and Study Selection

**Figure 1:**
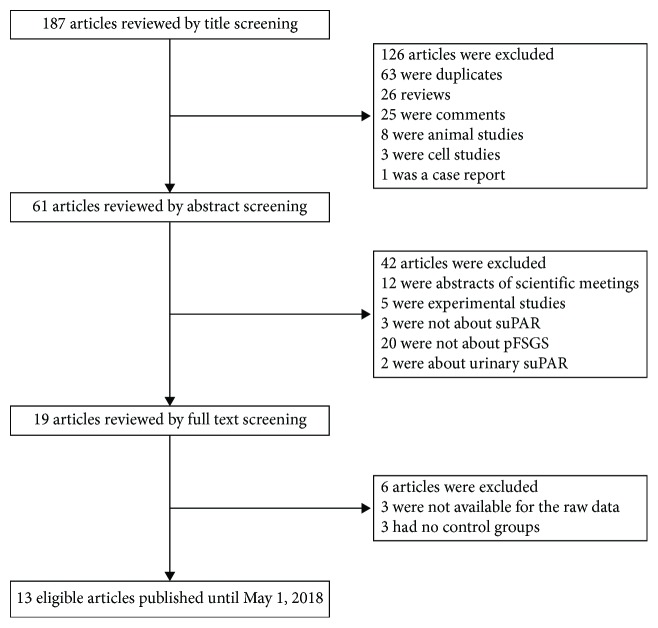
Flow chart of literature search. ^∗^Abbreviations: SUPAR: soluble urokinase-type plasminogen activator receptor; pFSGS: primary focal segmental glomerulosclerosis.

We performed a PubMed and EMBASE search to identify eligible articles. A forward search of the retrieved articles was performed, and “Google Scholar” was also assessed to screen for nonindexed publications. The last search in EMBASE and PubMed was performed on May 1, 2018. The search terms included focal segmental glomerulosclerosis OR FSGS AND soluble urokinase-type plasminogen activator receptor OR urokinase plasminogen OR suPAR. Records were managed by EndNote X8.0 (Clarivate Analytics, Philadelphia, PA, United States) to remove duplicates. Publications were screened first by title, second by abstract, and finally by full text, based on our eligibility criteria (Figure 1).

### 2.2. Inclusion and Exclusion Criteria

We included cross-sectional or longitudinal studies which compared serum suPAR levels in patients with pFSGS and healthy controls or non-FSGS glomerular diseases: minimal change disease (MCD), membranous nephropathy (MN), and immunoglobulin A nephropathy (IgAN). Studies which compared suPAR levels of active FSGS (defined as initial diagnosis or during relapse) with FSGS during remission were also included. We excluded studies that have measured suPAR levels in the urine or other body fluids. The exclusion criteria also included review articles, case reports, and animal experiments.

### 2.3. Data Extraction and Outcomes

Data extraction was carried out as recommended by the Cochrane handbook and included authors, year of publication, study design, participants, demographic characteristics, histopathology, and measurement of serum suPAR. Both review of full texts and extraction of data were independently performed by two reviewers (Lee JM and Yang JW). Any disagreement between the two primary reviewers was resolved by discussion with the third party (Shin JI).

Serum suPAR levels were collected as mean ± standard deviation (SD). Where data were given in median and interquartile ranges (IQR), we used the quantile method for estimating *X* (mean) and *S* (standard deviation) from mean and IQR, proposed by Wan and colleagues [17]:

(1) $$\begin{array}{c} \bar{X}\approx \frac{q_{1}+m+q_{3}}{3},\\ S\approx \frac{q_{3}-q_{1}}{1.35}. \end{array}$$

### 2.4. Quality Assessment

This meta-analysis was conducted and reported according to the PRISMA (Preferred Reporting Items for Systematic Reviews and Meta-Analysis) statement (Supplementary Table S1). Risk of bias of individual studies at the outcome level was assessed by using the Newcastle-Ottawa Scale (NOS) (Supplementary Table S2).

### 2.5. Statistical Analysis and Evaluation of Heterogeneity and Publication Bias

In the meta-analysis, the standardized mean difference (SMD) method and corresponding 95% confidence intervals (CIs) were used to compare suPAR levels. Random effect models were used because of heterogeneity of the included studies. We assessed the heterogeneity of the studies by using the Cochran *Q* test, and a *P* value of <0.1 was considered significant. The inconsistency across the studies was also measured by the *I* ^2^ metric, as a measure of the percentage of total variation across the studies because of the heterogeneity. *I* ^2^ values of <25, 25-75, and >75% were considered to represent low, moderate, and high levels of heterogeneity, respectively. Publication bias of each article was estimated by inspecting the funnel plot and using the Egger test when there were 10 or more eligible studies. All analyses were conducted using Comprehensive Meta-Analysis v.2.0 (Biostat, Englewood, NJ, USA) and RevMan 5.3 (The Nordic Cochrane Centre).

## 3. Results

### 3.1. Study Selection and Characteristics

A total of 187 articles were identified using electronic and manual research. After reviewing titles and abstracts, 19 studies were selected for full-text reading. Of them, 6 were excluded (3 studies had no control groups and 3 studies were not available for the raw data) to finally include 13 eligible articles [13–15, 18–27]. The detailed process of article selection is shown in Figure 1. The respective characteristics of the included studies are described in detail in Table 1.

The PRISMA checklist for meta-analysis is shown in Supplementary Table S1. The study quality assessed by using the Newcastle-Ottawa scale (NOS) scored 6 in nine studies and 7 in four studies (range, 1 (very poor) to 9 (very high); Supplementary Table S2).

### 3.2. Meta-Analysis of suPAR Levels in pFSGS Patients Compared to Healthy Controls

**Figure 2:**
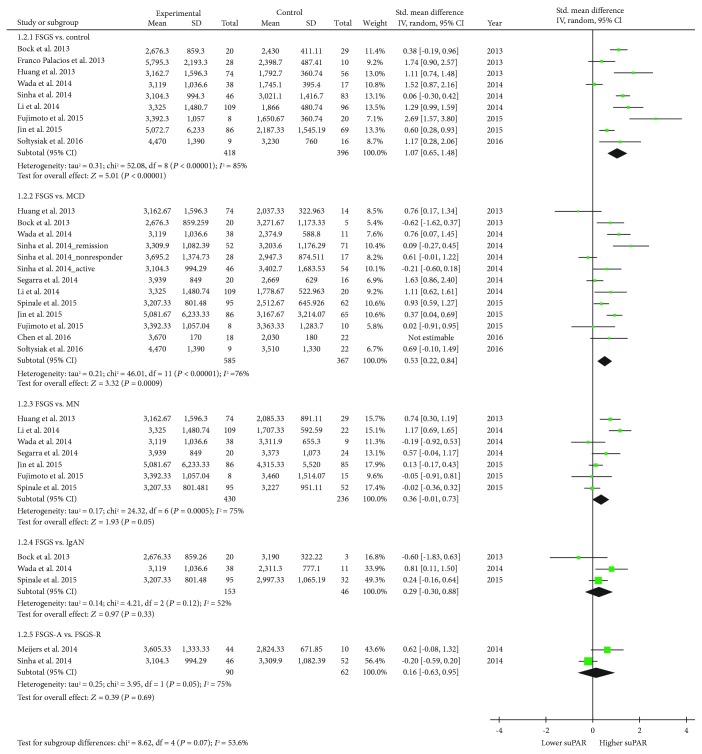
Forest plot of random effects meta-analysis of serum suPAR levels in FSGS patients. Squares are proportional to study weight. ^∗^Abbreviations: SUPAR: soluble urokinase-type plasminogen activator receptor; FSGS: focal segmental glomerulosclerosis.

**Figure 3:**
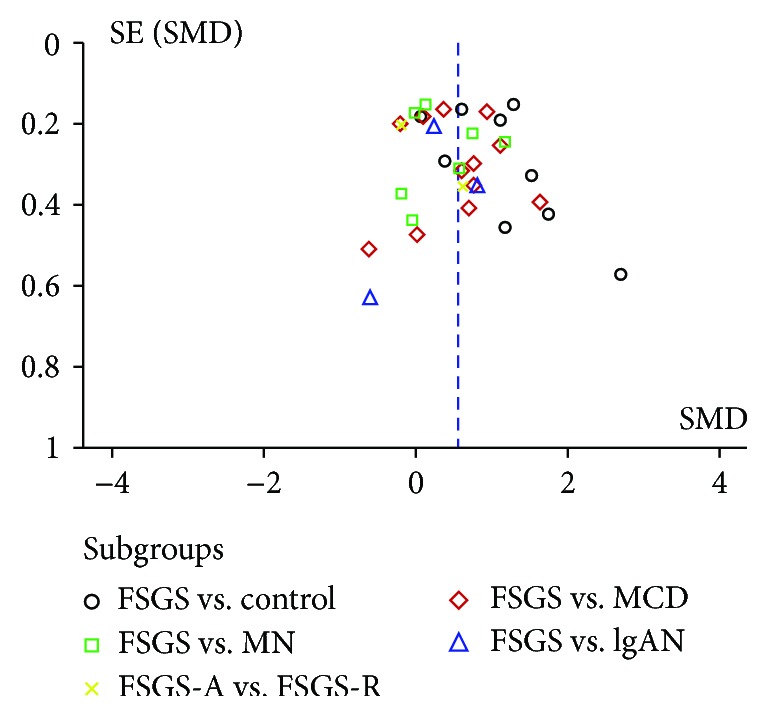
Funnel plot of standard error in meta-analysis of serum suPAR levels in pFSGS patients compared with controls. ^∗^Abbreviations: SUPAR: soluble urokinase-type plasminogen activator receptor; pFSGS: primary focal segmental glomerulosclerosis.

A meta-analysis on pFSGS patients and healthy controls was performed. Among the 13 studies, there were 9 studies which examined suPAR levels in 418 pFSGS patients and 396 healthy controls. The results revealed that suPAR levels were significantly higher in the pFSGS group compared with those in the control group (SMD 1.07, 95% CI 0.65 to 1.48; participants = 814; studies = 9; *I* ^2^ = 85%) (Table 2; Figure 2). The overall mean concentration of serum suPAR was 4470 ± 1390 (pg/mL) in pFSGS groups and 2399 ± 487 (pg/mL) in the control group (Table 2). A funnel plot of standard error for this meta-analysis did not reveal significant publication bias (Figure 3).

### 3.3. Meta-Analysis of suPAR Levels in pFSGS Patients Compared to Disease Controls

Ten studies compared suPAR levels in 503 patients with pFSGS and 296 with MCD, indicating significantly higher levels in patients with pFSGS compared to those with MCD, 3550 ± 1456 pg/mL and 2790 ± 1048 pg/mL (SMD 0.53, 95% CI 0.22 to 0.84; participants = 952; studies = 13; *I* ^2^ = 85%) (Table 2; Figure 2). In this meta-analysis, the study by Sinha et al. [14] was counted separately for three times because they compared serum suPAR levels in three different groups: patients at active (nephrotic) state, patients in remission, and nonresponders.

Patients with pFSGS were compared with MN and IgAN patients for their serum suPAR levels. The results were statistically insignificant for FSGS (3604 ± 1865 pg/mL) versus MN (3069 ± 1600 pg/mL) (SMD 0.36, 95% CI -0.01 to 0.73; participants = 666; studies = 7) and FSGS (3001 ± 899 pg/mL) versus IgAN (2833 ± 722 pg/mL) (SMD 0.29, 95% CI -0.30 to 0.88; participants = 199; studies = 3).

### 3.4. Meta-Analysis of suPAR Levels in pFSGS Patients with and without Active Proteinuria

We compared serum suPAR levels in pFSGS patients with active proteinuria (*n* = 90) and those in remission of proteinuria (*n* = 62). The meta-analysisshowed that there was a trend towards higher suPAR levels during active disease which, however, did not yield significance (SMD 0.29, 95% CI -0.30 to 0.88; participants = 199; studies = 3) (Table 2; Figure 2).

### 3.5. Serum suPAR Levels in Pediatric and Adult Groups

**Figure 4:**
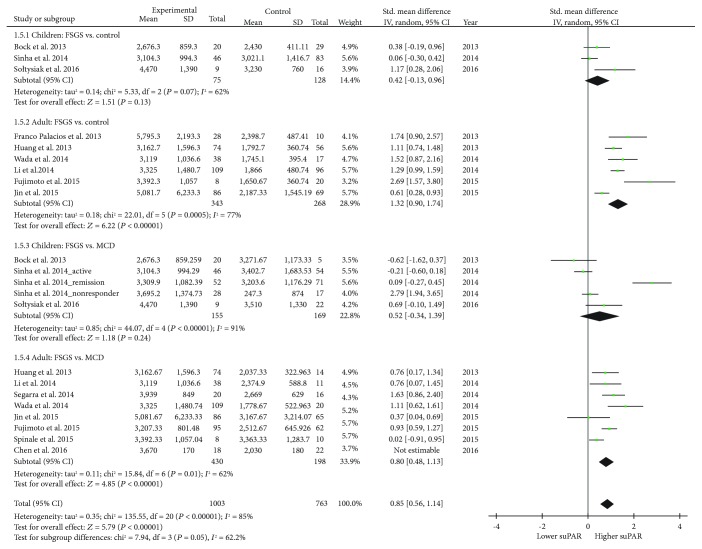
Forest plot of meta-analysis of serum suPAR levels compared in children and adult groups. ^∗^Abbreviations: SUPAR: soluble urokinase-type plasminogen activator receptor; pFSGS: primary focal segmental glomerulosclerosis; MCD minimal change disease.

We compared serum suPAR levels in pediatric and adult groups. The results revealed that children with pFSGS had no significant difference in their suPAR levels compared to any controls (Figure 4; Table 3). For adult patients, however, serum suPAR levels were significantly more elevated in pFSGS patients compared to both healthy controls and MCD controls. It showed statistically powerful results even after eliminating the outliers. In the comparison meta-analysis on adults with FSGS vs. MCD (1.5.4 of Figure 4), the study by Chen et al. [26] was counted as an outlier and not included for the final calculation. Basal serum levels of suPAR in both healthy and MCD controls were higher in children than adults (Table 3).

### 3.6. Assessment of Heterogeneity and Publication Bias

We assessed statistical heterogeneity between the included studies (Table 2). Since the *I* ^2^ test showed a value > 50%, indicating substantial heterogeneity, we used random effect models for meta-analyses. The funnel plot showed near symmetry (Figure 2).

## 4. Discussion

The usefulness of suPAR as a biomarker of FSGS has been a controversial issue. In an *in vitro* model, Alfano et al. showed that suPAR induces downmodulation of nephrin in human podocytes and that it may result in renal dysfunction in different human pathologies characterized by increased concentration of suPAR [28]. Elevation of suPAR levels was demonstrated in patients with pFSGS as well. Wei and colleagues, in their comprehensive study, reported a marked elevation of suPAR levels in two large cohorts, 84.3% (North American) and 55.3% (the European PodoNet) of pFSGS patients compared with 6% of controls [11]. Such an observation was consistently noted when compared to patients with other glomerulonephritis [10, 11, 13, 15, 29]. Serum suPAR levels correlated with proteinuria and declined estimated glomerular filtration rate (eGFR) [11, 13]. Furthermore, suPAR has been demonstrated to correlate with development of recurrent FSGS after kidney transplantation (KT) [30].

However, other investigators found that serum suPAR levels in FSGS were similar to controls and questioned its significance as a biomarker and that suPAR failed to discriminate pFSGS from other glomerulonephritis forms such as MCD, MN, IgAN, lupus nephritis, or nonglomerular CKD [3, 14, 18–23, 31–34]. Moreover, elevated serum suPAR levels have been demonstrated in patients with nonrenal conditions, such as cancer, sepsis, systemic inflammatory response syndrome (SIRS), and cardiovascular disease, and have been shown to be associated with a poor clinical outcome [35–37]. In addition, it has been suggested that the inverse correlation between suPAR and eGFR may be due to impaired renal excretion itself, rather than its function as a biomarker [14]. The results from these studies implied that while suPAR may indeed be increased in pFSGS, it might be nonspecifically involved in the pathogenesis of various diseases.

We propose the following explanations for pleiotropic effects of suPAR:

First, different isoforms and glycosylation statuses of suPAR may have different impacts. suPAR is present in variable forms from suPAR I to III. Trachtman et al. considered that it is likely that while all forms of suPAR can bind to *α*v*β*3 integrin which is a key modulator in the pathogenic process in podocytes, its subsequent activation might vary depending on the specific form of suPAR [38]. Moreover, Maas et al. speculated that the vitronectin-binding capacity of suPAR fragments might determine the activity as a FSGS factor [16]. In addition, the glycosylation status of suPAR may influence inducing proteinuria in pFSGS [39]. Since the currently available ELISA kit can only detect glycosylated suPAR of full length [39], characterization of the different isoforms and their biologic activity should later be addressed [3].

Second, modifying factors may have mediated suPAR-induced activation of *α*v*β*3 integrin. Loss of podocyte-protective factors or an underlying permissive genetic background has been proposed [3, 40]. Recently, SMPDL-3b was reported as an important regulator of suPAR-induced activation of *α*v*β*3 integrin signaling in podocytes by Yoo et al. [41].

Third, major confounders of suPAR need to be controlled. In normal populations, higher suPAR levels were found in women, smokers, older subjects, and African race [3, 42, 43]. These factors are required to be matched or adjusted when selecting healthy or disease controls. Furthermore, serum levels of inflammation markers (i.e., C-reactive protein (CRP) or erythrocyte sedimentation rate (ESR)) and renal function (GFR) need to be adjusted for, as inflammation itself can affect suPAR and low GFR may amplify suPAR levels due to impaired clearance [3].

Fourth, with regard to statistics, most of the studies performed simple comparison analysis presenting the differences in suPAR levels among groups. A more powerful study would require performing multiple logistic regression analysis to find independent predictors and receiver operating characteristic (ROC) curve analysis to calculate sensitivity and specificity [3].

In the present meta-analysis, serum suPAR levels were elevated in pFSGS patients compared to controls and this finding was consistent when compared to MCD, but not for MN or IgAN. We postulate that the results may still imply a potential role of suPAR in differential diagnosis of pFSGS from other forms of nephrotic glomerulonephritis, since distinguishing FSGS from MCD has long been a diagnostic challenge. However, we also noticed that serum suPAR levels had no significant difference in children and that we also found that serum FSGS levels were generally elevated overall in children than in adults. This finding may be biased due to the absolute paucity of pediatric data or have been affected by elevated basal suPAR levels in pediatric controls. In either case, we propose that future studies on serum suPAR should better be age-stratified.

Moreover, the results must be interpreted with caution for the following reasons. First, pFSGS is a heterogeneous disorder with diverse etiopathogenesis and different histopathology. Sometimes, even misclassification may occur since the distinction between primary and secondary FSGS may not always be feasible [3]. Since the etiology has a wide spectrum, it is important to specify the subgroup of patients in whom suPAR is the contributing circulating factor [44]. It has also been suggested that the role of suPAR should be interpreted in the context with recurrent FSGS after KT, which is considered to be circulating factor-mediated [3]. In short, the effect of circulating factors, such as suPAR on pFSGS, may have a different impact depending on the etiopathology. Second, there is still paucity of data. The present meta-analysis contained 13 studies and even fewer for subgroup analysis. Further subgroup comparisons including histopathology, gender, age, and ethnicity groups were not available. In addition, although there were studies that reported the correlation of serum suPAR with eGFR [13, 14, 19, 20, 22, 27], subgroup analyses by eGFR were not feasible because the data were not differentially present according to eGFR. Further meta-analyses containing studies with more patients would help in verifying the results of the present study.

Our study has some limitations. There were a few (three) studies excluded from the full-text research due to lack of raw data. In addition, some studies did not present the mean ± standard deviation (SD), hampering the meta-analysis. Also, there remains the possibility of existing case reports or series that were not accessible. In future studies, an individual patient data meta-analysis and propensity score matching would be powerful in investigating whether suPAR could be a reliable surrogate biomarker of pFSGS.

Although the results require cautious interpretation, the study provides evidence based on current publications. The present meta-analysis showed that serum suPAR was increased in patients with pFSGS compared to controls and also differentially among other glomerulonephritis forms. suPAR levels did not differ between pFSGS patients with active disease and those in remission. Further studies are needed to investigate its clinical usefulness as a biomarker.

## Figures and Tables

**Table 1:** Characteristics of all studies included in the meta-analysis.

Author, year	Age group	Study groups	Estimated GFR (ml/min per 1.73 m^2^)	*N*	Gender M/F	Age (mean ± SD or range)	suPAR levels (pg/ml)
Bock et al., 2013 [18]	Children	Control	N/A	29	N/A	N/A	2430 ± 411
		pFSGS	81.9 ± 47.3	20	12 : 8	12.1 ± 5.0	2676 ± 859
		MCD		5	N/A	N/A	3272 ± 1173
		IgAN		3	N/A	N/A	3190 ± 322

Huang et al., 2013 [15]	Adult + children	Control	Graphs only	56	33 : 23	21-47	1793 ± 361
		pFSGS		74	50 : 24	13-84	3163 ± 1596
		MCD		14	7 : 7	17-71	2037 ± 323
		MN		29	18 : 11	33-79	2085 ± 891

Franco Palacios et al., 2013 [19]	Adult	Control	Inverse correlation with eGFR (*R* = −0.36, *P* = 0.003)	10	4 : 6	42.6 ± 9.6	2399 ± 487
		pFSGS		28	N/A	51.2 ± 11.2	5795 ± 2193

Wada et al., 2014 [20]	Adult	Control	Inverse correlation with eGFR (*R* ^2^ = 0.242, *P* < 0.001)	17	9 : 8	45.3 ± 15.5	1745 ± 395
		pFSGS		38	26 : 12	55.6 ± 16.3	3119 ± 1037
		MCD		11	6 : 5	41.2 ± 18.1	2375 ± 589
		MN		9	4 : 5	67.9 ± 10.3	3312 ± 655
		IgAN		11	5 : 6	42.2 ± 20.8	2311 ± 777

Sinha et al., 2014 [14]	Children	Control	95.6 ± 25.4	83	42 : 41	8.3 ± 4.1	3021 ± 1417
		pFSGS-A	105.3 ± 34.8^a^	46	83:37^a^	9.4 ± 4.8^a^	3104 ± 994
		pFSGS-N	Inverse correlation with eGFR (*P* < 0.001)	28			3695 ± 1374
		pFSGS-R		52	a		3310 ± 1082
		MCD-A		54	85:32^b^	7.8 ± 4.3^b^	3403 ± 1684
		MCD-N		17			2947 ± 875
		MCD-R		71			3204 ± 1176

Li et al., 2014 [13]	Adult	Control	125 ± 21	96	73 : 23	28 ± 8	1866 ± 481
		pFSGS	100 ± 31	109	83 : 26	28 ± 14	3325 ± 1481
		MCD	No correlation with eGFR	20	17 : 3	19 ± 6	1779 ± 523
		MN		22	19 : 3	40 ± 19	1707 ± 593

Meijers et al., 2014 [21]	Adult	pFSGS-A	62.5 (36.8–98.7)	44	31 : 13	47 (33 − 60)	3605 ± 1333
		pFSGS-R	57.7 (47.2–92.4)	10	5 : 5	43 (39 − 70)	2824 ± 672

Segarra et al., 2014 [22]	Adult	pFSGS	Inverse correlation with eGFR (*r*: –0.467, *P* < 0.001)	20	11 : 9	52.6 ± 16.2	3939 ± 849
		MCD		16	6 : 10	34.5 ± 18.6	2669 ± 629
		MN		24	16 : 8	53.7 ± 12.2	3373 ± 1073

Spinale et al., 2015 [23]	Adult + children	pFSGS		95	64 : 31	36 (17 − 52)	3207 ± 801
		MCD		62	36 : 26	14 (6 − 25)	2513 ± 646
		MN		52	32 : 20	54 (41 − 61)	3227 ± 951
		IgAN		32	19 : 13	42 (32 − 54)	2997 ± 1065

Fujimoto et al., 2015 [24]	Adult	Control		20	15 : 5	29.5 (25.5 − 34.0)	165 ± 361
		pFSGS		8	4 : 4	48 (29 − 68)	3393 ± 1057
		MCD		12	7 : 5	47 (33.5 − 61.0)	3363 ± 1284
		MN		15	11 : 4	66 (60.8 − 71.3)	3460 ± 1514

Jin et al., 2015 [25]	Adult	Control		69	39 : 30	35 (20 − 46)	2187 ± 1545
		pFSGS		86	48 : 38	32 (16 − 78)	5082 ± 6233
		MCD		65	34;31	39 (18 − 69)	3168 ± 3214
		MN		85	50 : 35	51 (34 − 75)	4315 ± 5520

Chen et al., 2016 [26]	Adult	pFSGS		18	14 : 4	56.83 ± 8.29	3670 ± 170
		MCD		22	19 : 3	36.00 ± 4.25	2030 ± 180

Sołtysiak et al., 2016 [27]	Children	Control	Inverse correlation with eGFR	16	N/A	13.4 ± 2.5^c^	3230 ± 760
		pFSGS	(*r*: –0.643, *P* not given)	9	N/A		4470 ± 1390
		MCD		22	N/A		3510 ± 1330

^a^Data for all patients with FSGS; ^b^data for all patients with MCD; ^c^data for all participants in this study. ^∗^Abbreviation used: SUPAR: soluble urokinase-type plasminogen activator receptor; pFSGS: primary focal segmental glomerulosclerosis; pFSGS-A: active primary FSGS in remission; pFSGS-R: pFSGS in remission; MCD: minimal change disease; MN: membranous nephropathy; IgAN: immunoglobulin A nephropathy; *N*: number; N/A: not available; SD: standard deviation.

**Table 2:** Summary of all meta-analysis data comparing primary FSGS with healthy and disease controls.

Group comparison	No. of studies	No. of subjects		suPAR levels (pg/ml)		Meta-analysis			Heterogeneity		
						Std diff in means	95% CI		*I* ^2^ (%)	Tau^2^	*P*
pFSGS vs. Controls	9	pFSGS 418	Controls 396	pFSGS 4470 ± 1390	Controls 2399 ± 487	1.07	0.65	1.48	85	0.31	<0.001
pFSGS vs. MCD	13	pFSGS 603	MCD 389	pFSGS 3550 ± 1456	MCD 2790 ± 1048	0.53	0.22	0.84	76	0.21	0.0009
pFSGS vs. MN	7	pFSGS 430	MGN 236	pFSGS 3604 ± 1865	MN 3069 ± 1600	0.36	-0.01	0.73	75	0.17	0.0005
pFSGS vs. IgAN	3	pFSGS 153	IgAN 46	pFSGS 3001 ± 899	IgAN 2833 ± 722	0.29	-0.30	0.88	52	0.14	0.33
pFSGS-A vs. FSGS-R	2	pFSGS-A 90	pFSGS-R 62	pFSGS-A 3355 ± 1164	pFSGS-R 3067 ± 877	0.16	-0.63	0.95	75	0.25	0.05

^∗^Abbreviations used: SUPAR: soluble urokinase-type plasminogen activator receptor; pFSGS: primary focal segmental glomerulosclerosis; pFSGS-A: active primary FSGS in remission; pFSGS-R: pFSGS in remission; MCD: minimal change disease; MN: membranous nephropathy; IgAN: immunoglobulin A nephropathy. ^∗^ *P* values were all two-tailed. Hedges' *g*, random effect.

**Table 3:** Comparison of pediatric and adult data.

	Serum suPAR levels (No. of studies/No. of patients)			
	pFSGS	Controls	pFSGS	MCD
Pediatric	3417 ± 1082 (3 studies, *n* = 75)	2894 ± 863 (3 studies, *n* = 128)	3451 ± 1140 (5 studies, *n* = 155)	3267 ± 1248 (5 studies, *n* = 169)
Adult	3979 ± 2266 (6 studies, *n* = 343)	1940 ± 605 (6 studies, *n* = 268)	3612 ± 1653 (8 studies, *n* = 448)	2492 ± 923 (8 studies, *n* = 220)

^∗^suPAR: soluble urokinase-type plasminogen activator receptor; pFSGS: primary focal segmental glomerulosclerosis; MCD: minimal change disease.

## Data Availability

The raw data supporting this meta-analysis are from previously reported studies and datasets, which have been cited. The processed data are included within the article. The full processed data in detail are also available from the corresponding author upon request.

## Supplementary Materials

Supplementary Materials Table S1: PRISMA 2009 checklist. Table S2: the Newcastle-Ottawa Scale (NOS).
